# Ericoid mycorrhizal fungi as biostimulants for improving propagation and production of ericaceous plants

**DOI:** 10.3389/fpls.2022.1027390

**Published:** 2022-11-16

**Authors:** Xiangying Wei, Wenbing Zhang, Faisal Zulfiqar, Chunying Zhang, Jianjun Chen

**Affiliations:** ^1^ Fujian Key Laboratory on Conservation and Sustainable Utilization of Marine Biodiversity, Fuzhou Institute of Oceanography, College of Geography and Oceanography, Minjiang University, Fuzhou, China; ^2^ Department of Horticultural Sciences, Faculty of Agriculture and Environment, The Islamia University of Bahawalpur, Bahawalpur, Pakistan; ^3^ Shanghai Engineering Research Center of Sustainable Plant Innovation, Shanghai Botanical Garden, Shanghai, China; ^4^ Mid-Florida Research and Education Center, Department of Environmental Horticulture, Institute of Food and Agricultural Sciences, University of Florida, Apopka, FL, United States

**Keywords:** biostimulants, blueberry, ericaceous plants, ericoid mycorrhiza, *Oidiodendron* *maius*, rhododendron

## Abstract

The mutualistic relationship between mycorrhizal fungi and plant roots is a widespread terrestrial symbiosis. The symbiosis enables plants to better adapt to adverse soil conditions, enhances plant tolerance to abiotic and biotic stresses, and improves plant establishment and growth. Thus, mycorrhizal fungi are considered biostimulants. Among the four most common types of mycorrhizae, arbuscular mycorrhiza (AM) and ectomycorrhiza (EcM) have been more intensively studied than ericoid mycorrhiza (ErM) and orchidaceous mycorrhiza (OrM). ErM fungi can form symbiotic relationships with plants in the family Ericaceae. Economically important plants in this family include blueberry, bilberry, cranberry, and rhododendron. ErM fungi are versatile as they are both saprotrophic and biotrophic. Increasing reports have shown that they can degrade soil organic matter, resulting in the bioavailability of nutrients for plants and microbes. ErM fungi can synthesize hormones to improve fungal establishment and plant root initiation and growth. ErM colonization enables plants to effective acquisition of mineral nutrients. Colonized plants are able to tolerate different abiotic stresses, including drought, heavy metals, and soil salinity as well as biotic stresses, such as pathogen infections. This article is intended to briefly introduce ErM fungi and document their beneficial effects on ericaceous plants. It is anticipated that the exploration of this special group of fungi will further improve our understanding of their value of symbiosis to ericaceous plants and ultimately result in the application of valuable species or strains for improving the establishment and growth of ericaceous plants.

## Introduction

Plant biostimulants are referred to as natural-occurring or synthetic substances, which when applied to soil, seeds, and/or plants, can promote plant growth. There are several definitions for plant biostimulants. du Jardin (2015) defined “a plant biostimulant is any substance or microorganism applied to plants with the aim to enhance nutrition efficiency, abiotic stress tolerance and/or crop quality traits, regardless of its nutrient content”. Yakhin et al. (2017) proposed that a biostimulant is “a formulated product of biological origin that improves plant productivity as a consequence of the novel or emergent properties of the complex of constituents, and not as a sole consequence of the presence of known essential plant nutrients, plant growth regulators, or plant protective compounds.” Under the new regulation of the European Union (EU, 2019), “a plant biostimulant shall be an EU fertilizing product, the function of which is to stimulate plant nutrition processes independently of the product’s nutrient content with the sole aim of improving one or more of the following characteristics of the plant or the plant rhizosphere: (1) nutrient use efficiency, (2) tolerance to abiotic stress, (3) quality traits, or (4) availability of confined nutrients in the soil or rhizosphere”. To be more explicit, plant biostimulants are products that can improve plant growth.

Biostimulants are derived from a wide range of biological and inorganic materials (Calvo et al., 2014) and have been classified into seven categories (du Jardin, 2015): (1) humic and fulvic acids or humic substances, which are a group of heterogeneous, highly acidic compounds resulting from the decomposition of soil animal, microbial, and plant residues; (2) protein hydrolysate and other nitrogen (N)-containing compounds, referring to a mixture of amino acids and peptides obtained through enzymatic and chemical hydrolyses of animal wastes and plant residues; (3) seaweed extracts and botanicals, which are derived from seaweed or plants with biostimulant activities; (4) chitosan and other biopolymers, this includes biodegradable and biocompatible poly- and oligomers produced from natural produces; (5) inorganic compounds, mainly referring to beneficial elements, such as silicon, titanium, sodium, selenium, iodine, and aluminum (Al); (6) beneficial fungi, which include those either associated, endophytic or symbiotic with plants that possess biostimulant activities; and (7) beneficial bacteria, also referring to associated, endophytic, or symbiotic bacteria promoting plant growth. Based on the above classifications, biostimulants could be broadly divided into non-microbial and microbial plant biostimulants (Rouphael and Colla, 2020). It was estimated that biostimulant products could grow to $2 billion in sales in 2018 (Calvo et al., 2014). More than 700 scientific papers on biostimulants had been published from 2009 to 2019 (Rouphael and Colla, 2020), indicating an increasing awareness of their importance to crop production.

Beneficial fungi include *Trichoderma* species, mycorrhizal fungi, yeasts, endophytes, and avirulent/hypovirulent strains of some pathogens (Ghorbanpour et al., 2018). Mycorrhizal fungi are a heterogeneous group of taxa that can establish symbiotic relationships with roots of most terrestrial plants, promoting plant acquisition of nutrients in exchange for carbon sources derived from photosynthesis (Addy et al., 2005; Bonfante and Genre, 2010; Leopold, 2016). Mycorrhizas are traditionally grouped into arbuscular mycorrhiza (AM), ericoid mycorrhiza (ErM), ectomycorrhiza (EcM), and orchidaceous mycorrhiza (OrM) (Pandey et al., 2019; Miyauchi et al., 2020). AMs are considered the most widespread symbiotic association as 80-90% of terrestrial plants can be colonized by this group of fungi (Gianinazzi et al., 2010). The beneficial effects of AMs on plants are known to be most noticeable when symbiotic relationships are established at the earliest stage of plant growth (Niemi et al., 2004). AM fungi have been reported as biostimulants (Xavier and Boyetchko, 2002; Rouphael et al., 2015), natural biofertilizers (Berruti et al., 2016), and plant growth regulators (Begum et al., 2019). However, ErM fungi as biostimulants have not been reported. An ErM is referred to as a symbiotic complex of plant roots and fungal components. ErM fungi represent a unique group of fungi that can symbolize with plants in the family Ericaceae or heather family and play vital roles for plants in adapting harsh growing environments (Read, 1996); however, ErM has been the least studied, and the least understood mycorrhizal symbiosis (Vohník, 2020).

**Figure 1:**
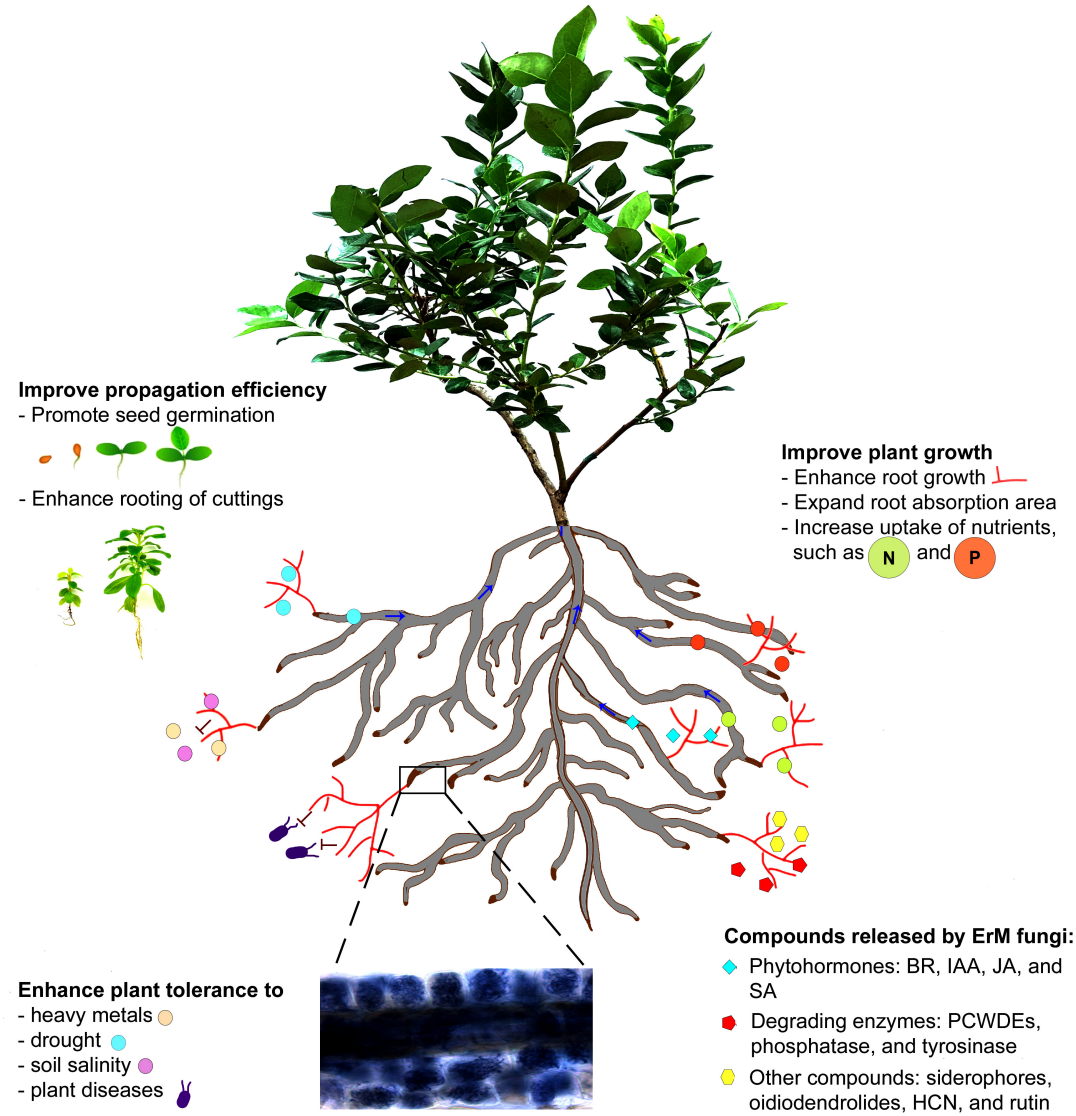
A schematic illustration of ericoid mycorrhizal fungi in establishment of the symbiotic relationship with an ericaceous plant (blueberry) and production bioactive compounds, which result in the improved seed germination and rooting of cuttings, and the symbiosis also enhances plant tolerance to different abiotic stresses and resistance to pathogen infections and promotes plant growth.

This article is intended to briefly introduce ErM fungi and review available literature related to ErM fungi in biosynthesizing bioactive compounds, promoting plant growth, and improving plant tolerance and resilience to abiotic and biotic stresses. Our review indicates that ErM fungi are valuable biostimulants that can synthesize bioactive compounds, including plant growth regulators, promote seed germination and rooting of cuttings, improve plant tolerance to heavy metals, drought, and soil salinity, and resistance to plant diseases, and enhance the growth of ericaceous plants (**Figure 1**).

## Ericoid mycorrhizal fungi

Ericoid mycorrhizal fungi largely belong to Ascomyceta and Basidiomycota (Perotto et al., 2018; Fehrer et al., 2019; Vohník, 2020). The most important ErM fungi in Ascomyceta include the *Hyaloscypha hepaticicola* aggregate, formerly known as the *Rhizoscyphus ericae* aggregate or *Hymenoscyphus ericae* aggregate (Fehrer et al., 2019), which include *H. hepaticicola*, possibly *Hyaloscypha variabilis* (syn. *Meliniomyces variabilis*) as well as *Oidiodendon maius* and *Leohumicola* spp (Vohník, 2020). *H. hepaticicola* was actually the first experimentally confirmed ErM species (Pearson and Read, 1973). *O. maius* was initially isolated by Barron (1962) from peat soils collected in Canada and subsequently from ericaceous plant roots in Japan (Tokumasu, 1973). Later, *O. maius* was identified from other plant roots and decayed organic materials, peat, and acidic soils (Rice and Currah, 2005; Rice and Currah, 2006). *Leohumicola* species are another group of ErM fungi, which have a remarkable tolerance to high temperatures (Adeoyo et al., 2018; Adeoyo et al., 2019).

The basidiomycetous ErM fungi are composed of sebacinoid fungi from Serendipitaceae (Vohník et al., 2016) and non-sebacinoid fungi (Kolařík and Vohník, 2018) as well as those from the *Kurtia argillacea* species complex (syn. *Hyphodontia argillaceum*, *Hyphoderma argillaceum*) (Vohník, 2020). With increasing exploitation of ErM fungi worldwide, more ericoid mycorrhizal fungi have been reported. For instance, two ascomycetous genera: *Cairneyella* (Midgley et al., 2016) and *Gamarada* (Midgley et al., 2018) were found to be ErM fungi in Australia.

Ericoid mycorrhizal fungi are able to colonize roots of ericaceous plants. The fungi initially grow on the surface of hair roots, establishing loose hyphal networks. Hyphae then penetrate cortical cell walls to form intracellular densely packed individual cells, which is known as coils (Vohník et al., 2012). Thus, ericoid mycorrhiza has structurally well-defined endomycorrhiza that are distinctly different from the other mycorrhizae due to the formation of fine compact intracellular hyphal coils in the rhizodermal cells of hair roots. The coil is the site for transferring nutrients absorbed by ErM fungi from the soil to root cells and carbohydrates fixed by plant photosynthesis to fungi. The hyphal sheaths or mantles around healthy hair roots are somewhat similar to those occurring in some EcM (Vohník, 2020). It was observed that both cell-to-cell (between neighboring rhizodermal cells) and single cell (from soil to individual rhizodermal cells) hyphal colonization happened in the rhizodermis of ErM-colonized hair roots (Massicotte et al., 2005; Vohník, 2020).

Interestingly, a recent study showed that ErM fungi, specifically those in the genus *Hyaloscypha* can colonize not only roots, but also stems, leaves, and flowers of *Vaccinium myrtillus* (Daghino et al., 2022). The colonization of the above-ground organs is explained by the evolutionary closeness between the genus *Hyaloscypha* and non-mycorrhizal fungal endophytes based on the genomic similarity. However, it is unknown at present what role *Hyaloscypha* plays in the aerial plant organs of ericaceous plants.

## Ericaceous plants and their growing conditions

The family Ericaceae has about 4,500 species across 125 genera, which are either herbs, dwarf shrubs, shrubs, or trees that are distributed in tundra, heathland, and understory of boreal forests in the Northern Hemisphere (Luteyn, 2002; Grelet et al., 2009). Economically important ericaceous plants include *Rhododendron* L., blueberry (*Vaccinium* sect. *Cyanococcus* Rydb. spp.), bilberry (*Vaccinium myrtillus* L.), cranberry (*Vaccinium* subg. *Oxycoccus* (Hill) A. Gray spp.), and huckleberry (*Vaccinium parvifolium* sm.). Plants in the genus *Rododendron* are popular ornamental plants. There are more than 28,000 cultivars of *Rhododendron* (12,989 azaleas, 14,298 rhododendron, and 108 azaleodendrons hybrids) in the International Rhododendron Registry held by the Royal Horticultural Society (Leslie, 2002). Additionally, rhododendron plants are important ethnopharmacological and toxicological plants (Popescu and Kopp, 2013; Wei et al., 2018). On the other hand, the berries, such as blueberry and cranberry have high nutraceutical and pharmaceutical value and are considered super fruit (Whyte and Williams, 2015; Vendrame et al., 2016). Blueberry varieties include lowbush, southern highbush, northern highbush, half-high, and rabbiteye. Among them, the production of highbush varieties increased substantially from 58,400 ha in 2007 to 110,800 ha in 2014. North America accounted for more than 50% of the production area, representing about 60% of highbush blueberry production in the world in 2014 (Qiu et al., 2018).

Ericaceous plants have some unique characteristics: (1) Roots are fibrous, very fine multicellular roots ranging from 100 to 750 μm in diameter, and cortical cells never form root hairs. Instead, roots of ericaceous plants are known as hair roots (Watkinson, 2016). Roots are mainly distributed in the upper 5 cm of soil depth, representing more than 50% of new roots produced during a growing season (Atucha et al., 2020). Root growth of cranberry exhibited a unimodal curve with one significant flush at bloom and a peak at the end of fruit maturation. (2) They are able to grow in soils low in pH (4 to 5) and poor in nutrient availability. Under such a pH range, nitrification could be largely negligible due to its detrimental effect on the nitrifying bacteria (Paul and Clark, 1989). It was proposed that ericaceous plants grown in the low pH soil might lose their ability to take up nitrate (NO_3_^-^). (3) Roots are colonized by mycorrhizal fungi, mainly ErM fungi (Xiao and Berch, 1992; Usuki et al., 2003; Vohník et al., 2007; Tian et al., 2011). Root cortex and epidermis could be fully filled with mycorrhizal hyphal coils (Atucha et al., 2020). The colonization plays a critical role for ericaceous plants to absorb nutrients including NO_3_^-^. Cranberry roots inoculated with *R. ericae* were able to absorb NO_3_^-^ under a low pH regime (Kosola et al., 2007). Accumulating evidence has indicated that the symbiosis established between roots and ErM fungi is essential for ericaceous plants to absorb nutrients and to survive and grow in the harsh environment (Read, 1996; Cairney and Meharg, 2003; Zhang et al., 2009; Perotto et al., 2012).

## Ericoid mycorrhizal fungi promote seed germination and rooting of cuttings

Ericoid mycorrhizal fungi have been shown to improve seed germination and rooting of microcuttings or stem cuttings of ericaceous plants (**Table 1**). Seeds of ericaceous plants are small with an average length of 1.5 mm and a mean width of 0.5 mm, and they generally have a poor germination rate due to the limited supply of nutrition from the endosperm. In a mesocosm study conducted for determining if novel ErM communities could assist or hamper the shift of northward species of *Rhododendron*, Mueller et al. (2022) reported that germination rates of *R. catawbiense* and *R. maximum* after inoculation with novel soils containing ErM fungi were significantly greater than those of controls (without mycorrhizal fungi) or inoculated with conspecific soils, 75.2% vs. 54.5% for *R. catawbiense* and 65.7% vs. 54.4% for *R. maximum*. The increased germination rates were attributed to the occurrence in ErM fungi, but the authors did not provide the underlying mechanisms. In addition to seed germination, ErM has been documented to promote seedling growth of *Rhododendron*. An ErM fungus known as *O. maius* Om19 inoculated in a peat-based substrate substantially enhanced seedling growth of *R. fortunei* Lindl. because root growth including root length, root numbers, root fresh weight as well as shoot growth, including shoot overall height and shoot fresh weight of Om19 colonized seedlings were doubled compared to the uninoculated control seedlings (Wei et al., 2016a). Additionally, overall fresh and dry weights of *R. fortunei* seedlings inoculated with Om19 were 81% and 84% higher than those of the control, respectively. Furthermore, genes related to N uptake and metabolism were analyzed by qRT-PCR, and results showed that the expression of an *ammonium transporter* (*AMT*), two *nitrate transporters* (*NRT1-*1 and *NRT1-2*), glutamate synthase (*GOGAT*), and glutamine synthetase (*GS*) were highly upregulated in plants inoculated with Om19, ranging from 2 to 9 folds greater than the uninoculated plants (Wei et al., 2016a).

**Table 1:** Ericoid mycorrhizal fungi improve seed germination and rooting of cuttings.

Fungal species	Host plant species	Observed responses	References
*Hymenoscyphus ericae* and *Pezizella ericae*	*Rhododendron minus* and *Rhododendron chapmanii*	Increased survival rates and subsequent growth	Barnes and Johnson, 1986
*Hymenoscyphus ericae*	*Vaccinium corymbosum* and *Calluna vulgaris*	Increased plantlet growth	Berta and Gianinazzi-Pearson, 1986
*Hymenoscyphus ericae*	*Pieris floribunda*	Stimulated microcutting growth *in vitro*	Starrett et al., 2001
*Oidiodendron griseum* and *Hymenoscyphus ericae*	*Leucothoe fontanesiana*	Increased root initiation and root growth of cuttings	Scagel, 2005b
*Oidiodendron maius*	*Vaccinium virgatum*	Two strains differentially altered root morphology cuttings	Baba et al., 2021a
*Oidiodendron maius*	*Vaccinium oldhamii*	Increased the length and branching of pioneer roots of seedlings	Baba et al., 2021b
*Oidiodendron maius*	*Rhododendron fortunei*	Enhanced rooting and root growth of microcuttings	Wei et al., 2020
Unspecified ericoid mycorrhizal fungi	*Rhododendron catawbiense* and *R. maximum*	Increased seed germination rates	Mueller et al., 2022

Ericoid mycorrhizal fungi can substantially improve rooting of stem cuttings as well as microcuttings derived from tissue culture (Eccher et al., 2010; Wei et al., 2020). Stem cuttings of blueberry plants inoculated with ErM fungi rooted more successfully and were followed by enhanced plant growth (Scagel et al., 2005a; Scagel et al., 2005b). Root initiation and root growth of dog hobble (*Leucothoe fontanesiana*) (Scagel, 2005b) and *Vaccinium meridionale*, a Colombian blueberry (Ávila Díaz-Granados et al., 2009) also increased with the inoculation of ErM fungi. Recently, micropropagation, shoot culture in particular, has been increasingly used for propagation of important ericaceous plants, such as rhododendron, cranberry, and blueberry (Fan et al., 2017; Wei et al., 2018). An interesting phenomenon observed during the rooting of microcuttings is that *in vitro* rooting is more difficult than *ex vitro* rooting (Gorecka, 1979; Fan et al., 2017; Qiu et al., 2018; Wei et al., 2018). To explore the underlying mechanisms behind this phenomenon, Wei et al. (2020) developed an *in vitro* culture system for *R. fortunei* and investigated the adventitious root (AR) formation in microcuttings inoculated with or without *O. maius* Om19. Key phytohormones and precursors involved in the pathway of indole-3-acetic acid (IAA) biosynthesis were analyzed in Om19 mycelium. Om19 was able to synthesize tryptophan (Trp), indole-3-pyruvate (IPA), and IAA, of which Trp concentration was greater than 4,000 mg/kg. The occurrence in Trp, IPA, and IAA indicated that Om19 biosynthesis of IAA is through the Trp-dependent pathway (Mano and Nemoto, 2012). Other hormones synthesized by Om19 include brassinolides (BRs), jasmonic acid (JA), and salicylic acid (SA). BRs were reported to positively impact the symbiosis of either tomato or tobacco roots with an AM fungus (von Sivers et al., 2019). JAs are known as a wound signal in AR formation because wounding quickly induces JA accumulation in plant tissues (Zhang et al., 2019). Increased JA concentrations have been shown to promote AR formation in cuttings by IAA accumulation in the base of stem cuttings towards AR source cells (Druege and Franken, 2019). SA is known for triggering systemic-acquired resistance (SAR) in plants (Chen et al., 1996). Low concentrations of SA was reported to promote AR formation and change the root apical meristem architecture, but high SA concentrations suppressed the root growth process (Pasternak et al., 2019). After Om19 inoculation, ARs rapidly appeared from microcuttings. Meanwhile, genes related the symbiosis including *SymRK* and *DMI* were activated in ARs, resulting in Om19 colonization of the roots. Furthermore, *YUC3*, a key gene controlling IAA biosynthesis in plants (Cheng et al., 2007; Won et al., 2011), genes encoding N absorption (*AMT* and *NRT*) and N metabolism (*GOGAT* and *GS*) as well as phosphate transporter (*PHT*) in Om19-inoculated plants were upregulated by 3 to 7 folds compared to control plants without Om19 inoculation. As a result, inoculated plants were able to take up significantly higher quantities of nutrients including N, P, K, Ca, Mg, and S, and plant growth substantially increased compared to the control plants. A working model for the Om19-mediated AR formation was proposed. The rapid AR formation on the one hand was induced by IAA produced by Om19 and on the other hand by IAA biosynthesized by plants. The high concentration of Trp synthesized by Om19 could be readily used by plant as the precursor to synthesis of IAA. The formation ARs, in turn, provided Om19 with host for colonization. This study for the first time documented the ability of Om19 to biosynthesize several hormones, of which IAA plays an important role in inducing AR formation.

This model also provides explanations as to why *ex vitro* rooting of microcuttings is more effective than *in vitro* rooting. A major component of commercial substrates is peat moss, ranging from 30% to 75% based on volume (Chen et al., 2005). As mentioned before, *O. maius* was initially isolated from peat soils by Barron (1962). Commercial substrates rich in peat moss might have ErM mycorrhizal fungi. Gorman and Starrett (2003) screened ErM occurrence in commercial substrates and found that the majority of the peat and peat-based substrates used in the U.S. and Canada contained ErM fungi. ErR fungi were reported to naturally colonize roots of blueberry plants during nursery production (Scagel, 2005a). Thus, the ErM fungi in the substrates could act similar to Om19 in biosynthesis of phytohormones including IAA for inducing AR formation of ericaceous plant cuttings. Those ErM may also synthesize a large amount of Trp for plants to produce endogenous IAA. In general, endogenous hormones are more effective than exogenous application. Ismail et al. (2021) reported that bacterial and fungal endophytic metabolites significantly enhanced plant growth compared to exogenous applied hormones. IAA biosynthesized inside plants by either plants or ErM could be considered endogenous and thus could be more effective for inducing AR formation than exogenous applied auxin during *in vitro* rooting.

Ericoid mycorrhizal fungi also produce other bioactive compounds (**Table 2**). Some can increase bioavailability of soil mineral elements, such as siderophore for chelating soil Fe (Ahmed and Holmström, 2014). Some are enzymes, including cell-wall-degrading enzymes (PCWDEs) that can degrade soil organic matter (SOM) (Kohler et al., 2015; Martino et al., 2018). Others have antimicrobial activities (Hosoe et al., 1999; Ouyang, 2021), and still others are antioxidants, such as rutin (Lin et al., 2021).

**Table 2:** Bioactive compounds released by ericoid mycorrhizal fungi.

Fungal species	Bioactive compounds	Role of the compound	References
*Cryptosporiopsis* sp.	Rutin	Antioxidative activity	Lin et al., 2021
*Hymenoscyphus ericae* and *Oidiodendron griseum*	Hydroxamate siderophore	A chelator product for improving iron bioavailability in soils	Schuler and Haselwandter, 1988
*Hymenoscyphus ericae*	Phenol-oxidation and extracellular o-polyphenol oxidase (tyrosinase)	Degraded lignin or soluble phenolic compounds	Bending and Read, 1997
*Hymenoscyphus ericae*	Chitinase	Involved in chitin degradation	Kerley and Read, 1997; Bougoure and Cairney, 2006
*Leohumicola incrustata*	Amyloglucosidase	Hydrolyzed individual glucose units from the non-reducing ends of starch chains	Adeoyo et al., 2018
*Oidiodendron* cf. truncatum	Four new tetranorditerpenoids, oidiodendrolides A, B, and C, and oidiodendronic acid	Antibiotic activity against pathogenic yeast	Hosoe et al., 1999
*Oidiodendron maius*	Plant cell wall-degrading enzymes, PCWDEs	Degraded plant cell wall	Kohler et al., 2015
*Oidiodendron maius*	Mucilage and soluble and wall-bound pigments	Chelated heavy metal ions	Martino et al., 2000b
*Oidiodendron maius*	Tryptophan, indole-3-pyruvate, indole-3-acetic acid (IAA), brassinolides (BRs), jasmonic acid (JA), and salicylic acid (SA)	Precursor for IAA biosynthesis. IAA, BRs, JA, and SA are plant growth regulators	Wei et al., 2020
*Oidiodendron flavum*	Harzianic acid	Antimicrobial activity	Ouyang, 2021
*Oidiodendron truncatum*	Fourteen norditerpene and three anthraquinone metabolites.	Antifungal activity	Rusman et al., 2020
Ericoid mycorrhizal fungi	Indole-3-acetic acid (IAA), hydrogen cyanide (HCN), siderophores, and phosphatase	IAA is plant growth regulator; HCN is a co-product of ethylene biosynthesis; siderophores improve nutrient bioavailability; and phosphatase solubilizes insoluble forms of phosphorus	Hamim et al., 2019

## Ericoid mycorrhizal fungi enhance plant growth

Ericoid mycorrhizal fungi have been well documented for promoting the growth of ericaceous crops. ErM fungi are able to establish symbiotic relationships with plant roots. The established symbiosis enables plants to better adapt to acidic soils with low pH values and low nutrient status and improve root acquisition of nutrients, thus, enhancing plant growth. The growth enhancement is attributed to several factors: (1) The ability of ErM to biodegrade SOM resulting in the bioavailability of nutrients for plants; (2) the symbiosis-resultant expansion of nutrient acquisition surface area for capturing more mineral elements; and (3) the upregulation of gene expression, such as those associated with symbiosis and N and P uptake and metabolism, leading to the increased N and P absorption and metabolism and enhanced plant growth.

### ErM fungi mediated degradation of soil organic matter

A unique characteristic of ErM fungi is their capability to biodegrade SOM (Martino et al., 2018). SOM is the organic fraction of the soil consisting of animal and plant residues and microorganisms at different stages of decomposition and contributes significantly to soil fertility and productivity (Myers and Leake, 1996; Biswas and Kole, 2017). Ericaceous plants are native to acidic soils, low in nutrients but high in recalcitrant organic materials, which results in a low bioavailability of mineral elements, particularly N and P (Cairney and Meharg, 2003). ErM fungi can degrade SOM, leading to the release of nutrients for host plants. Among the ErMs, *R. ericae* and *O. maius* were found to degrade cellulose, tannic acid, pectin, and chitin (Rice and Currah, 2001; Thormann et al., 2002; Rice and Currah, 2005). A wide range of enzymes were released from the two ErM fungi for degrading fungal and plant cell wall polymers, complex aliphatic compounds, and organic phosphorus (Smith and Read, 2008). Kohler et al. (2015) sequenced 13 EcM, ErM, and OrM species as well as five saprotrophs and found that EcM fungi have reduced numbers of genes for PCWDEs compared to their ancestral wood decayers. Later, Martino et al. (2018) sequenced genomes of ErM fungi *R. ericae*, *Meliniomyces bicolor*, *M. variablilis*, and *O. maius* and compared their gene repertoires with EcM, OrM, and six pathogenic or saprotrophic Leotimycetes and 50 other Basidiomycetes and Ascomycetes. The authors found that ErM fungi possessed lipases, polysaccharide-degrading enzymes, proteases, and enzymes in secondary metabolism that were comparable to those of pathogens and saprotrophs but higher than those of EcM fungi, suggesting the ErM fungi are unique due to their dual saprotrophic and biotrophic lifestyle. Additionally, RNA-Seq analysis showed that highly upregulated genes in ErM fungi were those involved in lipases, cell wall-degrading enzymes (CWDEs), transporters, proteases, and mycorrhizal-induced small-secreted proteins (MiSSPs). Thus, ErM fungi represent a unique group of fungi in degradation of SOM, which explains in part as to why ericaceous plants can survive and grow in the acidic and low nutrient soils.

Soil organic nitrogen (ON) is a fraction of SOM, including intracellular and cell-wall bound proteins and nucleic acids of plant and microbial origin (Nemeth et al., 1987; Abuarghub and Read, 1988). The main groups of soil ON compounds are aliphatic-N, including polysaccharide N and amino-N as well as aromatic N, such as those present in soil humus (Chen and Xu, 2006; Talbot and Treseder, 2010). These large molecules can be degraded by enzymes of ErM fungi into monomers, such as oligomer, small peptides, and amino acids (Talbot and Treseder, 2010) which are small enough for soil microbes and plants to take up or further mineralize and incorporate as ammonium and nitrate (Séneca et al., 2021). Studies have shown that mycorrhizal fungi and plant roots can take up amino acids. ErM fungi *R. ericae* were able to use all 20 common amino acids except glycine (Leake and Read, 1990; Cairney et al., 2000; Midgley et al., 2006), while *O.* spp. can use alanine, glutamic acid, arginine, lysine, proline, asparagine, glutamine, histidine, and cysteine (Whittaker and Cairney, 2001). Furthermore, ErM fungi have the capacity to use proteins, peptides or even chitin as a sole N source (Leake and Read, 1990; Read, 1996). Lin et al. (2011) evaluated three ErM isolates, Rf9 and Rf32 belonging to the genus *Cryptosporiopsis* and Rf28, a member of *Phialocephala* in decomposition SOM. Both Rf28 and Rf32 possessed the highest decomposition rates, up to 10.4% in 70 days, but Rf9 had a decomposition rate of 6.8%. Enzymatic assay showed that Rf28 and Rf 32 secreted cellulase, laccase, peroxidase, and tyrosinase, but Rf9 mainly released peroxidase and tyrosinase. Seedlings of *R. formosanum* Hemsl. grown in substrate without ErM inoculation showed chlorotic symptoms and limited root growth, while those inoculated with ErM exhibited much stronger growth vigor than those of the control. This study implies that ErM-mediated decomposition of soil ON provides seedlings with nutrients, particularly N.

### Enhanced growth of plants through symbiosis

Inoculation of ErM fungi can substantially enhance the growth of ericaceous plants. In general, ericaceous plants can be naturally colonized by ErM fungi (Hambleton and Currah, 1997; Gorman and Starrett, 2003), but the colonization rates are low, less than 15%. Inoculation of ErM fungi can increase colonization rates, up to 30% (Scagel, 2005a), and the colonization promoted growth in blueberry cultivars. **Table 3** lists growth enhancement of ErM fungi on ericaceous plants. Inoculation of ErM fungi affected acclimatization and growth of microcuttings of *Pieris floribunda* (Starrett et al., 2001). Container-grown *R. indica* inoculated with *O. maius* Om19 had more abundant roots and a larger above ground canopy than those uninoculated (Wei et al., 2016a). Plant height, root, shoot, and total fresh weights of seedlings of *R. kanehirae* inoculated with two ErM strains significantly increased when ammonium was used as a N source (Lin et al., 2021). ErM fungi in nature Finnish peat moss promoted rooting of rabbiteye blueberry (*V. virgatum* Ait.) and vegetative growth (Li et al., 2021). Dry weights of shoots, leaves, and roots of rooted cuttings were 2.37, 4.51, and 4.34 g, respectively compared to 0.47, 0.90, and 0.67 g of those grown in sterilized peat moss. Furthermore, root P and Mg concentrations and shoot K content increased in plants grown in unsterilized peat moss. A recent study also showed that single inoculation of *V. corymbosum* with *O. maius* or *Phialocephala fortinii* significantly increased plant dry weight (Wazny et al., 2022).

**Table 3:** Ericoid mycorrhizal fungi improve rooting of propagules and enhance plant growth.

Fungal species	Host plant species	Observed responses	References
*Cryptosporiopsis* sp	*Rhododendron pseudochrysanthum*	Enhanced seedling growth as total fresh weight of inoculated seedlings was higher than the control seedlings	Lin et al., 2021
*Hymenoscyphus ericae*	*Pieris floribunda*	Increased survival rate of micropropagated plants during *ex vitro* acclimatization.	Starrett et al., 2001
*Hymenoscyphus ericae*, *Oidiodendron griseum*, and *Pezizella ericae*,	Seven highbush blueberry cultivars (*Vaccinium corymbosum*)	Improved plant growth as inoculants increased plant growth; but root/shoot biomass ratios decreased due to the application of organic or inorganic fertilizers	Scagel, 2005a; Scagel, 2005b
*Hymenoscyphus ericae* and *Oidiodendron griseum*	*Vaccinium corymbosum*	Improved plant growth as inoculated plants produced significantly larger floral displays, more fruits per inflorescence, and heavier fruits with lower sugar content, than uninoculated control plants.	Brody et al., 2019
*Meliniomyces variabilis*, *Oidiodendron maius*, *O. or Rhizoscyphus ericae*	*Vaccinium virgatum* ‘Rabbiteye blueberry Ait.	Promoted vegetative growth including more leaves and shoots, greater total leaf area and shoot length than those grown in sterilized substrate	Li et al., 2021
*Oidiodendron maius* and *Hymenoscyphus* sp.	*Vaccinium corymbosum*	Improved plant growth and vitality and increased biomass accumulation	Wazny et al., 2022
*Oidiodendron maius*	*Rhododendron* cv. Azurro	Increased root biomass and plant phosphorus concentrations	Vohník et al., 2005
*Oidiodendron maius*	*Rhododendron fortunei*	Enhanced plant growth evidenced by larger canopies and root systems of seedlings, and genes related to plant uptake of N and N metabolism were highly upregulated	Wei et al., 2016a; Wei et al., 2016b
*Oidiodendron maius*	*Arabidopsis thaliana*	Increased shoot and root biomass, shortened the primary root and increased the lateral root length and number of seedlings	Casarrubia et al., 2016
*Oidiodendron maius*	*Rhododendron kanehirae*	Promoted plant growth since plant height, roots, shoots, and total fresh weight significantly increased when ammonium was used as a N source	Lin et al., 2020
*Oidiodendron maius*	*Rhododendron fortunei*	Enhanced microcutting growth and increased N and P contents of plants	Wei et al., 2020
*Oidiodendron maius*	*Vaccinium virgatum* Ait ‘Tifblue’	Altered the length and branching of pioneer and/or fibrous roots of rooted cuttings	Baba et al., 2021a

The increased growth of plants is largely attributed to the following factors: (1) The bioavailability of small ON compounds degraded by ErM fungi mentioned above. (2) The symbiosis results in the establishment of root and hyphal networks that greatly expand root surface areas for capturing more mineral nutrients. Atucha et al. (2020) studied the phenology of roots, root anatomy and morphology in terms of root orders in cranberry (*V. macrocarpon* Ait). More than 50% of new roots produced during the growing season were vertically distributed in the upper 5 cm of soil depth, and mycorrhizal fungi were able to colonize the intact cortex and epidermis of the first three root orders. It is known that large root systems generally have a larger root surface areas and concomitantly shorter average half distance between root axes in the soil or substrate for more effectively capturing nutrient elements (Chen and Gabelman, 2000). Thus, absorption of N, P, and other mineral nutrients was markedly increased in ErM colonized plants. (3) ErM fungi are able to biosynthesize and release hormones for stimulating root growth, such as IAA, BR, JA, and SA, which can stimulate plant growth and improve plant stress tolerance. (4) ErM-colonization results in the increased expression of a large number of genes. To gain insight into the intimate relationships of ErM fungi with ericaceous plants and the mechanism underlying growth stimulation, Wei et al. (2016b) analyzed transcripts induced by the symbiosis between ErM Om19 and *R. fortunei* using RNA-Seq. The symbiosis induced 16,892 upregulated genes in Om19-coloinzed roots. Homologous to symbiosis related genes, such as *SymRK*, *CCaMK*, *DM1*, *NORK*, genes involved in N uptake including *AMT3*, *NRT1-1*, *NRT1-2*, as well as N metabolism, such as *GS-1* and *GS-2* and *GOGAT-1* and *GOGAT-2* were highly upregulated in roots inoculated with Om19, suggesting that ErM fungi might share the same strategy as AM fungi in the establishment of symbiotic relationships with ericaceous plants. The increased expression of the genes in N uptake and metabolism corresponded to the increased N absorption and metabolism as well as plant growth. Thus, the ability of ericaceous plants to survive and grow in acidic and low nutrient soils is largely associated with their symbiotic relationship with ErM fungi (Cairney and Meharg, 2003).

It is worthy of note that different strains within an ErM species may perform differently in regulation of N uptake in ericaceous plants. Five strains isolated from *Calluna vulgaris* had different capacities to use organic and mineral N sources (Grelet et al., 2005). Different isolates of epacrid root endophytes differed in the use of amino acids (Whittaker and Cairney, 2001). Using ^15^N tracing, Grelet et al. (2009) found that the rates of appearance of ^15^N in shoots of cranberry after roots were inoculated with three strains of Helotiales were low and not different from the control (no ErM inoculation) when nitrate was used as a N source. However, differences occurred among strains when glutamine was used as a N source. When NH_4_ was used a N source, two strains had the highest rates compared to the other strains. Thus, for practical application of ErM fungi as biostimulants, appropriate species or strains should be chosen.

## Improve plant tolerance to abiotic and biotic stresses

Plants as sessile organisms and permanently stay in their established sites. In addition to harsh environmental conditions, ericaceous plants often encounter other stressful factors, such as salt, drought, heavy metals as well as plant pathogens. Evidence has shown that in addition to growth promotion, symbiotic ErM fungi can substantially improve plant tolerance to abiotic and biotic stresses (**Table 4**). The enhanced stress tolerance in turn can improve plant productivity compared to those without symbiotic establishment.

**Table 4:** Ericoid mycorrhizal fungi improve plant tolerance to abiotic stresses.

Fungal species	Host plant species	Observed responses	References
*Hymenoscyphus ericae*	*Calluna vulgaris* and *Vaccinium macrocarpon*	Improved plant tolerance to Fe by limiting its transport to shoots	Shaw et al., 1990
*Hymenoscyphys ericae*	*Vaccinium macrocarpon*	Reduced Fe and Mn in leaves of mycorrhizal plants and increased Fe and Mn in root tissues, protected shoots from metal stress on the basis of exclusion rather than accumulation	Hashem, 1995a; Hashem, 1995b
*Hymenoscyphus ericae*	*Vaccinium mocrocarpon*	Provided plants with organic and inorganic P	Myers and Leake, 1996
*Hymenoscyphus ericae*	*Calluna vulgaris*	Enhanced plant tolerance to arsenate	Sharples et al., 2000a; Sharples et al., 2000b
*Hymenoscyphus complex*	*Woollsia pungens*	Improved plant tolerance to drought	Chen et al., 2003
*Meliniomyces variabilis* and *Oidiodendron maius*	*Rhododendron groenlandicum*, *Vaccinium myrtilloides*, and *Vaccinium vitisidaea*	Increased plants tolerance to salt stress	Fadaei et al., 2020
*Oidiodendron maius*	*Vaccinium corymbosum*	Improved plant tolerance to Al by restriction of Al in roots	Yang and Goulart, 2000
*Oidiodendron maius*	*Vaccinium myrtillus*	Enhanced plant tolerance to Zn by producing extracellular compounds to chelate Zn	Martino et al., 2000a; Martino et al., 2000b
*Oidiodendron maius*	*Rhododendron fortunei*	Increased N bioavailability to seedlings and induced the expression of genes related to N uptake and metabolism	Wei et al., 2016b
*Pezoloma ericae*	*Vaccinium macrocarpon*	Increased the ability of plant to utilize NO_3_−-N	Kosola et al., 2007

### Resistance to heavy metals

Another distinct characteristic of ErM fungi is their adaptability to heavy metal stress (Meharg and Cairney, 2000). *C. vulgaris* is a dominant plant in mine spoil sites as it can tolerate Cu^2+^ and Zn^2+^. Such a tolerance is attributed to its symbiotic relationship with ErM fungi. In the early 1980s, Bradley et al. (1981) reported that resistance of *C. vulgaris* to Cu^2+^ and Zn^2+^ was constitutive even in those mycorrhizal endophyte plants collected from sites without metal contamination (Bradley et al., 1981; Bradley et al., 1982). Martino et al. (2000b) reported that two isolates of *O. maius* from metal contaminated soils showed enhanced tolerance to Zn^2+^ *in vitro*, and later they reported that the mechanism behind the tolerance was due to the production of extracellular compounds, such as malate and citrate either to solubilize or chelate heavy metal ions (Martino et al., 2003). Subsequently, the ErM metal tolerant isolate known as *Oidiodendron maius* Zn has been used as model for studying the molecular basis underlying ErM-mediated metal tolerance (Daghino et al., 2016). Its genome was sequenced (Kohler et al., 2015). A recent report showed that a homeostatic mechanism at the cellular level mediated by transport proteins may play an important role (Ruytinx et al., 2020). Blueberry plants colonized by ErM fungi were also found to be able to tolerate high concentrations of Fe and Mn (Hashem, 1995a; Hashem, 1995b) as well as Al (Yang and Goulart, 2000). In addition to tolerance to Cu^2+^, Cd^2+^, and Zn^2+^, ErM fungi are particularly resistant to arsenate (AsO_4_^3-^). Sharples et al. (2001) compared As resistance between isolates of *Hymenoscyphus ericae* derived from *C. vulgaris* in soils contaminated with AsO_4_^3-^ and natural heathland soils and found that *H. ericae* isolated from the mine sites sustained significant growth at AsO_4_^3-^ concentration up to 4.67 mol m^–3^; however, the growth of the isolates from the heathland soils were almost completely inhibited. All isolates regardless of their origins had an identical response to Cu^2+^. These results suggest that *H. ericae* response to As is adaptive but their response to Cu^2+^ is constitutive.

Although some progress on the tolerance of ErM fungi to heavy metals has been made, our understanding on how ErM colonization can improve plant tolerance to heavy metal stress remains incomplete. Sharples et al. (2001) reported that *C. vulgaris* tolerance to As in the mine sites was due to the colonization of *H. ericae*, which allows the host to maintain an adequate supply of PO_3_^3-^ to limit the level of AsO_4_^3-^ in the cytosol, i.e., an ErM-mediated avoidance mechanism in *C. vulgaris.* To address ErM fungal colonization modulated plant tolerance to heavy metals, Casarrubia et al. (2020) studied *O. maius* mediated Cd tolerance in *V. myrtillus* and found that ErM colonized roots exposed to Cd had a reduced level of Cd compared to those uninoculated. Transcriptomic analysis showed that GSH metabolism was involved in the Cd tolerance as phytochelatins are biosynthesized from GSH, which can bind Cd to reduce toxicity (Chen and Goldsbrough, 1994; Chen et al., 1997). Some plant metal transporters were also regulated during the symbiosis and may be responsible for the reduced Cd content observed in mycorrhizal roots exposed to this metal.

### Tolerance to salt and drought stress

Several recent studies showed that ErM fungi can effectively improve ericaceous plant tolerance to salt stress. A recent study showed that velvetleaf blueberry (*V. myrtilloides*), labrador tea (*R. groenlandicum*), and lingonberry (*V. vitisidaea*) plants inoculated with *M. variabilis* had increased dry weights of roots when imposed on NaCl-treatment. Inoculation of *O. maius* increased root dry weight accumulation in lingonberry plants treated with NaCl at 30 mM (Fadaei et al., 2020). Salt stress generally disrupts plant water relations by adversely affecting water uptake and osmotic balance as well as root hydraulic conductivity in plants (Sutka et al., 2011), which then lead to the decrease in cell turgor and stomatal opening and the inhibition of cell elongation. As a result, transpiration rates and net photosynthetic rates decreased, and plant growth was suppressed (Vaziriyeganeh et al., 2018). In the report of Fadaei et al. (2020), transpiration and net photosynthetic rates of three ericaceous plant species treated with 30 mM NaCl were drastically reduced when grown in a substrate without ErM inoculation, but such detrimental effects were either completely or partially reversed by the inoculation of *O. maius* and *M. variabillis*. It was reported that EcM and AM colonization of roots increased the expression level of root aquaporins, resulting in the substantial improvement of root hydraulic conductivity (Xu et al., 2015). The authors believed that ErM fungi may act similar to AM in the alleviation of plant salt stress.

The ErM colonization also enhances drought tolerance of ericaceous plants. Mu et al. (2021) studied responses of lowland and upland blueberry seedlings to drought stress. Seedlings were grown in sterilized soil under controlled environmental conditions and inoculated with *M. variabilis*, *O. maius*, *P. ericae*, and *Pezoloma ericae*. All plants were imposed on three cycles of drought stress through withdrawing watering. Uninoculated, well-watered upland plants after three weeks of drought treatments produced higher dry weights compared to the uninoculated lowland plants. This difference, however, was offset after the plants were inoculated with ErM fungi, indicating that ErM addition significantly improved drought tolerance of the lowland and upland plants. Among the four ErM species, *Pezicula ericae* was found to be the most effective in enhancing drought resistance in lowland and upland seedlings as its inoculation maintained a higher water potential in plant shoots and higher net photosynthetic and transpiration rates, thus increased dry weight production.

### Responses to biotic stress

Information regarding ErM fungi improving ericaceous plant resistance to pathogens is rather limited. In a study conducted to test if ErM fungi could suppress the infection of soil-borne pathogens on mycorrhizal roots of *C. vulgaris* and *R. hirsutum*, Grunewaldt-Stöcker et al. (2013) inoculated *Pythium* spp. and *Phytophthora cinnamomic*, respectively to roots. The establishment of mycorrhizae, pathogen infections, and disease development in plants were examined microscopically. Results showed that ErM fungi suppressed the growth of external pathogenic mycelium and reduced pathogen infections. A complete reduction was observed at higher ErM colonization levels. However, pathogen infection occurred in those with low ErM colonization. These results showed that ErM colonization played a role in the direct suppression of oomycete pathogens from infection of ericaceous plants.

Systemic acquired resistance (SAR) is an immune response of plants against pathogen infection. It has been reported that some mycorrhizal fungi and plant growth promoting bacteria can induce SAR against a wide range of plant pathogens (Pieterse et al., 2014; Backer et al., 2018). However, SAR induction by ErM fungi has not been reported in ericaceous crops. Considering the fact that SA can effectively induce SAR in plants (Chen et al., 1996; Metraux, 2001) and *O. maius* can biosynthesize SA (Wei et al., 2020), it is likely that ErM fungi, *O. maius* in particular, should be able to induce SAR in plants, providing plants with long-lasting resistance to some plant pathogens. Further studies are warranted to test this hypothesis.

## Conclusion and future perspectives

Ericoid mycorrhizal fungi have saprotrophic and biotrophic lifestyles and are able to biodegrade SOM and establish symbiotic relationships with plants in the family Ericaceae. The degradation of SOM results in the bioavailability of nutrients to themselves and plants. The symbiosis extends their life cycle and also enhances seed germination, rooting of cuttings, plant growth as well as tolerance to abiotic and biotic stresses. This review documents that the improved plant growth is related to hormones produced by ErM fungi and also colonization-resultant expression of genes in N and P absorption and metabolisms. Thus, ErM fungi is considered biostimulants for promoting the establishment and growth of ericaceous plants. Considering some species in the family Ericaceae are economically important crops, it is expected that some ErM fungi could be developed as biofertilizers for improve plant propagation and production.

Our understanding of the ErM-mediated biostimulating effects on ericaceous plants, however, is largely incomplete. Further research is warranted to (1) identify and isolate key genes from ErM fungi in biodegradation of SOM, particularly ON and explore the genes for improving soil fertility, (2) evaluate important ErM species or strains in biosynthesis of hormones and isolate those for increased production of hormones through bioreactor and utilize the isolates for improving plant propagation and production, (3) analyze heavy metal tolerant species or strains of ErM through omics to identify the mechanism underlying metal tolerance and use the isolates for phytoremediation of metal-contaminated soils, and (4) develop ErM biofertilizers by combining different species or strains, each with specific biostimulating effects on propagation, growth promotion, and/or stress tolerance for improving the productivity of ericaceous plants.

## Author contributions

JC, XW, CZ, and FZ conceived the idea, WZ conducted literature search and prepared the tables and figures. JC and XW wrote the initial draft. All authors edited, refined the manuscript, and approved for its submission.

## Funding

This work was supported in part by the Natural Science Foundation of Fujian Province to XW (General program, Grant No. 2020J01867).

## Acknowledgments

The authors would like to thank Ms. Terri A. Mellich for critical review of this manuscript.

## Conflict of interest

The authors declare that the research was conducted in the absence of any commercial or financial relationships that could be construed as a potential conflict of interest.

## Publisher’s note

All claims expressed in this article are solely those of the authors and do not necessarily represent those of their affiliated organizations, or those of the publisher, the editors and the reviewers. Any product that may be evaluated in this article, or claim that may be made by its manufacturer, is not guaranteed or endorsed by the publisher.
